# Crocin Suppresses LPS-Stimulated Expression of Inducible Nitric Oxide Synthase by Upregulation of Heme Oxygenase-1 via Calcium/Calmodulin-Dependent Protein Kinase 4

**DOI:** 10.1155/2014/728709

**Published:** 2014-04-15

**Authors:** Ji-Hee Kim, Ga-Young Park, Soo Young Bang, Sun Young Park, Soo-Kyung Bae, YoungHee Kim

**Affiliations:** ^1^Department of Molecular Biology, College of Natural Sciences, Pusan National University, Jangjeon-dong, Keumjeong-gu, Busan 609-735, Republic of Korea; ^2^Department of Dental Pharmacology, School of Dentistry, Yangsan Campus of Pusan National University, Yangsan 626-870, Republic of Korea

## Abstract

Crocin is a water-soluble carotenoid pigment that is primarily used in various cuisines as a seasoning and coloring agent, as well as in traditional medicines for the treatment of edema, fever, and hepatic disorder. In this study, we demonstrated that crocin markedly induces the expression of heme oxygenase-1 (HO-1) which leads to an anti-inflammatory response. Crocin inhibited inducible nitric oxide synthase (iNOS) expression and nitric oxide production via downregulation of nuclear factor kappa B activity in lipopolysaccharide- (LPS-) stimulated RAW 264.7 macrophages. These effects were abrogated by blocking of HO-1 expression or activity. Crocin also induced Ca^2+^ mobilization from intracellular pools and phosphorylation of Ca^2+^/calmodulin-dependent protein kinase 4 (CAMK4). CAMK4 knockdown and kinase-dead mutant inhibited crocin-mediated HO-1 expression, Nrf2 activation, and phosphorylation of Akt, indicating that HO-1 expression is mediated by CAMK4 and that Akt is a downstream mediator of CAMK4 in crocin signaling. Moreover, crocin-mediated suppression of iNOS expression was blocked by CAMK4 inhibition. Overall, these results suggest that crocin suppresses LPS-stimulated expression of iNOS by inducing HO-1 expression via Ca^2+^/calmodulin-CAMK4-PI3K/Akt-Nrf2 signaling cascades. Our findings provide a novel molecular mechanism for the inhibitory effects of crocin against endotoxin-mediated inflammation.

## 1. Introduction

Inflammation is a defense response against infection, toxin exposure, or tissue injury. Inflammatory responses involve the infiltration of leukocytes to the site of infection or injury, and macrophages play an important role in regulation of inflammatory and immune responses through phagocytosis and secretion of proinflammatory mediator. In response to LPS, a major constituent of the Gram-negative bacterial cell wall, macrophages induce NF-*κ*B-dependent expression of inducible nitric oxide synthase (iNOS) and subsequently produce nitric oxide (NO). Overproduction of NO resulting from inappropriate activation of macrophages is implicated in the pathologies of many chronic inflammatory diseases such as rheumatoid arthritis, atherosclerosis, multiple sclerosis, and shock [1–3]. Therefore, the inhibition of NO production and suppression of mechanisms responsible for the activation of inflammatory responses are regarded as clinical strategies for the treatment of chronic inflammation.

Heme oxygenases (HOs) catalyze the rate-limiting step in oxidative degradation of cellular heme into carbon monoxide (CO), biliverdin, and free iron [4]. Two HO isoforms, HO-1 and HO-2, have been identified; HO-2 is constitutively expressed, while HO-1 is potently induced in many types of cells by numerous stress stimuli including oxidative stress, hypoxia, heavy metals, and cytokines. HO-1 and its enzymatic by-products provide a host defense mechanism that can protect the body against oxidative injury and also contribute to the anti-inflammatory activity of cells and tissues [5]. Previous studies suggested that, when macrophages are induced to overexpress HO-1 prior to stimulation with LPS, the subsequent proinflammatory response is markedly inhibited [5]. In activated macrophages, HO-1 expression or CO treatment inhibits the production of proinflammatory mediators such as nitric oxide (NO), prostaglandin E2 (PGE2), tumor necrosis factor-*α* (TNF-*α*), interleukin-1*β* (IL-1*β*), IL-6, monocyte chemoattractant protein-1, and macrophage inflammatory protein-1*β* [5–7]. Moreover, an increasing number of therapeutic agents have been reported to induce HO-1 expression and exert anti-inflammatory effects through HO-1 induction.

HO-1 is primarily regulated at the transcriptional level via signaling pathways involved in survival and stress responses. A redox-sensitive transcription factor NF-E2-related factor 2 (Nrf2) plays a central role in the inducible expression of HO-1. Under normal conditions, Nrf2 is sequestered in the cytoplasm by Kelch-like ECH-associated protein 1 (Keap1) and degraded by the ubiquitin-dependent 26S proteasome system [8]. Upon activation, Nrf2 released from Keap1 inhibition translocates to the nucleus, heterodimerizes with Maf, and binds antioxidant response elements (AREs) located in the promoter regions of many detoxifying/antioxidant genes, including HO-1 [9]. HO-1 expression is known to be promoted by phosphatidylinositol 3-kinase (PI3K)/Akt, as well as mitogen-activated protein kinases (MAPKs) including p38, c-Jun N-terminal kinase (JNK), and extracellular signal-regulated kinase (ERK). However, these signaling pathways leading to HO-1 expression vary depending on the type of cells and stimuli.

Crocin, crocetin digentiobiose ester, is a water-soluble carotenoid pigment found in the fruit of* Gardenia jasminoides* or stigma of* Crocus sativus* [10, 11]. These plants are primarily used in various cuisines as a seasoning and coloring agent, as well as in traditional medicines for the treatment of edema, fever, and hepatic disorder. A number of pharmacological studies have demonstrated that crocin has a wide range of activities including antioxidant [12–14], anti-inflammatory [15–17], anticancer [18], antiatherosclerotic, and hepatoprotective effects [13, 19–21]. In this study, we examined the molecular mechanism responsible for the anti-inflammatory effects of crocin. The results presented herein provide the first evidence that crocin induces HO-1 expression via Ca^2+^/calmodulin-dependent protein kinase 4 (CAMK4), which subsequently inhibits the lipopolysaccharide- (LPS-) induced iNOS expression in murine macrophages.

## 2. Materials and Methods

### 2.1. Reagents

**Figure 1:**
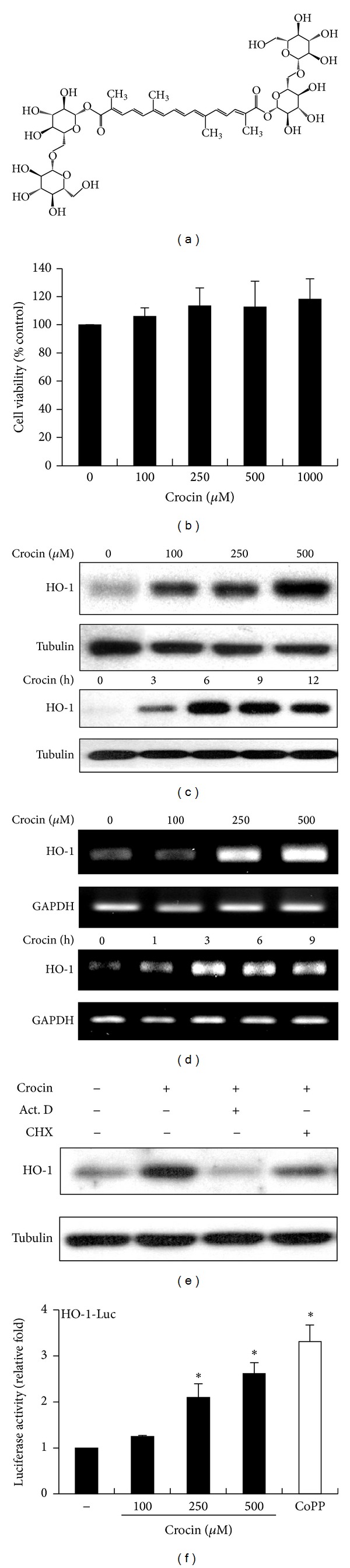
Induction of HO-1 by crocin. (a) Chemical structure of crocin. (b) RAW 264.7 cells were incubated with the indicated concentrations of crocin for 24 h, after which cell viability was measured by MTT assay. (c) RAW 264.7 cells were treated with the indicated concentrations of crocin for 6 h (upper panel) or with 500 *μ*M crocin for various periods (lower panel), and HO-1 protein levels were analyzed by Western blotting. (d) Cells were treated with the indicated concentrations of crocin for 3 h or with 500 *μ*M crocin for various periods and HO-1 mRNA levels were measured by RT-PCR. (e) Cells were treated with crocin (500 *μ*M) for 6 h in the presence of Act. D (1 *μ*g/mL) or CHX (1 *μ*g/mL), after which protein levels of HO-1 were measured by Western blotting. (f) Cells were transfected with HO-1 promoter-luciferase construct and then treated with the indicated concentration of crocin or CoPP (10 *μ*M). Equal amounts of cell extracts were then assayed for dual luciferase activity. *P < 0.05 versus the group treated with PBS (vehicle). GAPDH and tubulin were used as loading controls.

Crocin (Figure 1(a)), LPS (phenol extracted from* Salmonella enteritidis*), 3-(4,5-dimethylthiazol-2-yl)-2,5-diphenyltetrazolium bromide (MTT), actinomycin D (Act. D), cycloheximide (CHX), 2-(3,6-bis(acetyloxy)-2,7-dichloro-9H-xanthen-9-yl)benzoic acid acetoxymethyl ester (Fluo-3/AM), and other reagents were purchased from Sigma-Aldrich (St. Louis, MO, USA). Cobalt protoporphyrin (CoPP) and zinc protoporphyrin IX (ZnPP) were bought from Enzo Life Sciences, Inc. (Farmingdale, NY, USA). LY294002, PD98059, SP600125, and SB203580 were obtained from A.G. Scientific (San Diego, CA, USA). Nifedipine, 1,2-bis (*o*-aminophenoxy) ethane-N,N,N′,N′-tetraacetic acid tetra (acetoxymethyl) ester (BAPTA/AM), and calmidazolium chloride were acquired from Calbiochem (EMD Millipore, Billerica, MA, USA). KN92, KN93, and KN62 were purchased from Cayman Chemical (Ann Arbor, MI, USA) and Calbiochem.

### 2.2. Macrophage Culture

Murine macrophage RAW264.7 cells were maintained in Dulbecco's modified Eagle's medium (GIBCO, Gaithersburg, MD, USA) supplemented with glutamine (1 mM) and 10% FBS (GIBCO, Gaithersburg, MD, USA) at 37°C under 5% CO_2_.

### 2.3. Cell Viability Assay

The cytotoxicity of crocin was assessed by a microculture tetrazolium- (MTT-) based colorimetric assay. Briefly, MTT was added to each well of a 96-well plate containing cells (final concentration 62.5 *μ*g/mL). After incubation for 3 h at 37°C under 5% CO_2_, the supernatant was removed and the formed formazan crystals in viable cells were solubilized with 150 *μ*L of DMSO. The absorbance of each well was then read at 570 nm using a microplate reader.

### 2.4. Western Blot Analysis

Cells were washed with phosphate buffered saline (PBS) three times and then scraped off and lysed with lysis buffer (PBS with 1% Triton X-100 and 1% deoxycholate). The protein concentration of lysates was determined using Bradford reagent (Bio-Rad, Hercules, CA, USA), after which equal amounts of protein were separated electrophoretically using 10% SDS-PAGE (sodium dodecyl sulfate-polyacrylamide gel electrophoresis). The gel was then transferred to 0.45 *μ*m nitrocellulose paper and incubated with anti-iNOS, p65, HO-1, Nrf2, phospho-CAMK4, Akt, ERK1/2, JNK, TBP, HDAC antibodies (Santa Cruz Biotechnology, Santa Cruz, CA, USA), CAMK4, phospho-Akt, phospho-ERK1/2, phospho-JNK antibodies (Cell Signaling Technology, Beverly, MA, USA) or *α*-tubulin antibody (Bio Genex, Fremont, CA, USA), and secondary antibody and then detected by an enhanced chemiluminescence detection system according to the recommended procedure (GE Healthcare, Piscataway, NJ, USA).

### 2.5. Reverse Transcription- (RT-) Polymerase Chain Reaction (PCR)

Total cellular RNA was isolated using RNA spin mini RNA isolation kits (GE Healthcare, Piscataway, NJ, USA) according to the manufacturer's instructions. 1 *μ*g of total RNA was reverse-transcribed using* Maxime* RT PreMix (Intron Biotechnology, Korea) and anchored oligo-dT_15_-primers. PCR was then performed using a TaKaRa PCR Thermal Cycler Dice (TaKaRa Bio Inc., Japan). The primer sequences were as follows: HO-1-sense (5′-GTTGACGGACCCCAAAAGAT-3′), HO-1-antisense (5′-CCTCATCCTGGAAGGTCCAC-3′), GAPDH-sense (5′-AGGTGGTCTCCTCTGACTTC-3′), and GAPDH-antisense (5′-TACCAGGAAATGAGCTTGAC-3′).

### 2.6. Measurement of Nitrite Concentration

NO synthesis in cell cultures was measured by a microplate assay method. To measure nitrite, 100 *μ*L aliquots were removed from conditioned medium and incubated with an equal volume of Griess reagent (1% sulfanilamide/0.1%* N*-(1-naphthyl)-ethylenediamine dihydrochloride/2.5% H_3_PO_4_) at room temperature for 10 min. The nitrite concentration was then determined by measuring the absorbance at 540 nm with a Vmax 96-well microplate spectrophotometer (Bio-Rad, Hercules, CA, USA). Sodium nitrite was used as a standard.

### 2.7. Transient Transfection and Dual Luciferase Assay

RAW 264.7 cells were transfected with antioxidant-response element (ARE) reporter plasmid (Stratagene, La Jolla, CA, USA) or HO-1 promoter reporter plasmid (kindly provided by Dr. Norbert Leitinger at the University of Virginia, School of Medicine) [22] and Renilla luciferase control plasmid pRL-CMV (Promega, Madison, WI, USA) using FuGENE-6 reagent (Roche Applied Science, Penzberg, Germany) according to manufacturer's instruction. Sixteen hours after transfection, the cells were incubated with the indicated concentrations of crocin for 8 h. Luciferase activity was then assayed using a dual-luciferase assay kit (Promega) according to the manufacturer's instructions. Luminescence was measured with a GloMax 96 microplate luminometer (Promega). ARE luciferase activity was normalized to control Renilla luciferase expression. All transfection experiments were performed in duplicate in three independent experiments.

### 2.8. Interference of HO-1 or CAMK4

The siRNAs for HO-1 and CAMK4 were purchased from Santa Cruz Biotechnology (Santa Cruz, CA, USA), and cells were transfected with HO-1 siRNA or negative control siRNA using INTERFERin (Polyplus transfection, France). The cells were then incubated for 48 h until protein expression was detected.

### 2.9. Measurement of Intracellular Ca^2+^ Concentration

Changes in intracellular Ca^2+^ concentration were measured using Fluo-3 loaded cells as described by June and Moore  [23] with some modifications. For loading, the cells were incubated with 4 *μ*g/mL of Fluo-3/AM in cell-loading media (Dulbecco's PBS, DPBS, with Ca^2+^ and Mg^2+^ containing 1% FBS) at 37°C for 30 min and then washed three times with DPBS and resuspended in cell-loading medium (Ca^2+^-containing medium) or DPBS without Ca^2+^ and Mg^2+^ (Ca^2+^-free medium). After treatment with crocin or other reagents, cells were analyzed using a flow cytometer (Beckman Coulter, Miami, FL, USA) with excitation at 488 nm and emission at 535 nm. A total of 10,000 live cell events gated on forward and side scatter characteristics were acquired. Dead cells were excluded by gating out cells without Fluo-3 AM fluorescence. Data were expressed as a percentage of responding cells.

### 2.10. Statistical Analysis

All results were expressed as means ± SE. Each experiment was repeated at least three times. Statistical analysis was performed using the SPSS software to determine significant differences. We used one-way analysis of variance (ANOVA) followed by Tukey's post hoc test for comparison of three or more groups. A *P* < 0.05 was considered statistically significant.

## 3. Results

### 3.1. Crocin Induces HO-1 Expression in Murine Macrophages

To examine the effects of crocin on cell viability, murine macrophage RAW 264.7 cells were treated with various concentrations of crocin and an MTT-based cell viability assay was conducted. As shown in Figure 1(b), cell viability was not reduced in response to treatment with up to 1 mM of crocin.

To investigate whether crocin induces HO-1 expression in macrophages, murine macrophage RAW 264.7 cells were incubated with various concentrations of crocin. The HO-1 protein level was significantly increased by crocin in a dose-dependent manner and peaked at 6 h after treatment (Figure 1(c)). Crocin also induced HO-1 in thioglycollate- (TG-) elicited mouse peritoneal macrophages (see Supplementary Figures 1(A) and 1(B) in the Supplementary Material available online at http://dx.doi.org/10.1155/2014/728709). Moreover, the mRNA level of HO-1 increased in response to treatment with crocin in a dose-dependent manner, suggesting that crocin induces the transcription of HO-1 (Figure 1(d)). To confirm that crocin-induced HO-1 expression is mediated by transcription and translation, we used actinomycin D (Act. D) to inhibit DNA-dependent RNA polymerase, and cycloheximide (CHX) to inhibit ribosomal protein synthesis. Cotreatment with crocin and Act. D or CHX significantly reduced HO-1 expression (Figure 1(e)). Furthermore, HO-1 promoter activity also increased in dose-dependent manner in response to crocin (Figure 1(f)). Cobalt protoporphyrin (CoPP), an inducer of HO-1 [24], was used as a positive control. Taken together, these results suggest that crocin primarily upregulates HO-1 expression at the transcriptional level without damaging cells.

### 3.2. HO-1 Mediates Inhibitory Effects of Crocin on iNOS Expression and NF-*κ*B Activation in LPS-Stimulated Macrophages

**Figure 2:**
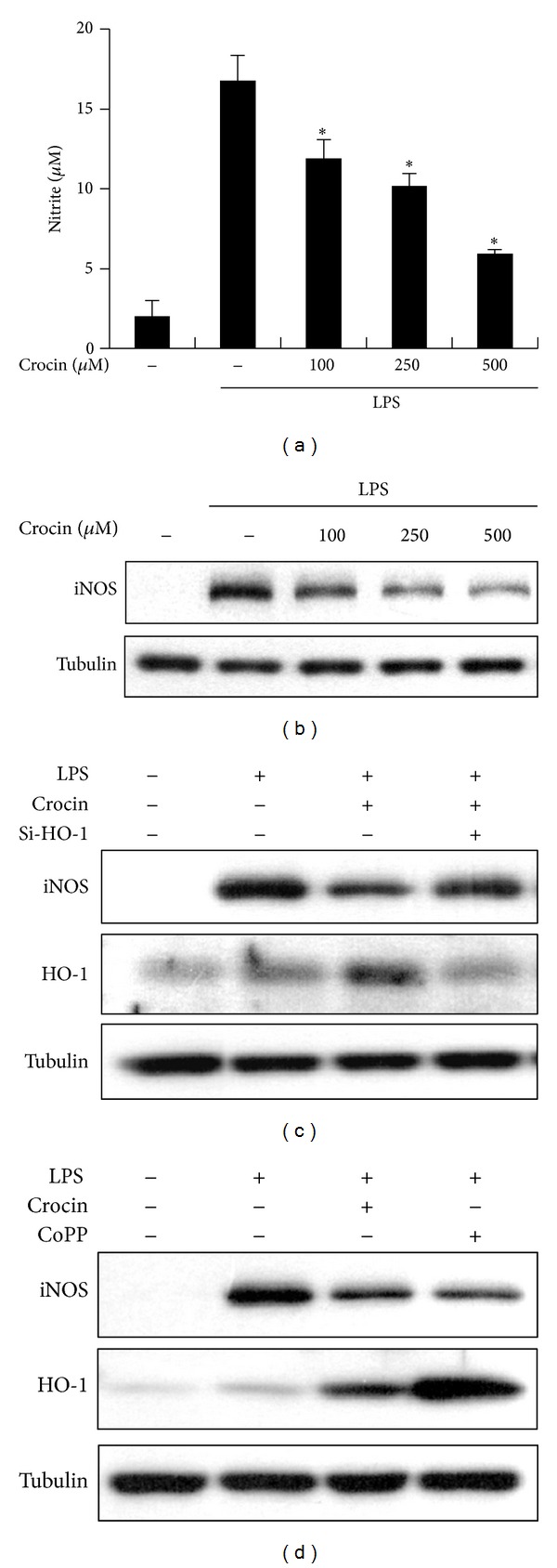
Inhibitory effects of crocin on NO production and iNOS expression in LPS-stimulated macrophages. ((a), (b)) RAW 264.7 cells were incubated with the indicated concentrations of crocin for 3 h and then stimulated with LPS (0.1 *μ*g/mL) for 24 h. Next, the supernatant was harvested and the amount of nitrite released from cells was measured by the Griess method (a). *P < 0.05 versus the group treated with LPS alone (no crocin). Equal amounts of cytosolic extract were analyzed by Western blotting (b). (c) Cells were transfected with HO-1 siRNA or control siRNA. Cells were treated with crocin (500 *μ*M) for 3 h and then were stimulated with LPS (0.1 *μ*g/mL) for 6 h. (d) Cells were incubated with crocin (500 *μ*M) or CoPP (10 *μ*M) for 3 h and then stimulated with LPS (0.1 *μ*g/mL) for 24 h, after which protein levels of iNOS and HO-1 were analyzed by Western blotting. Tubulin was used as a loading control.

To investigate the anti-inflammatory effects of crocin, we examined the effects of crocin on NO synthesis. The amount of LPS-induced NO released into culture supernatant was suppressed by crocin in a dose-dependent manner (Figure 2(a)). To determine whether the decreased NO synthesis was correlated with iNOS expression, we analyzed the amount of iNOS by Western blot analysis. The expression of iNOS was dramatically suppressed by crocin in a dose-dependent manner (Figure 2(b)). These results indicate that crocin inhibits NO release by affecting the iNOS expression level. Crocin also inhibited LPS-induced NO production and iNOS expression in TG-elicited mouse peritoneal macrophages (Supplementary Figures 1(C) and 1(D)). To determine whether crocin exhibits its anti-inflammatory effects through the induction of HO-1, we applied an HO-1 small interfering (si) RNA system to knock down HO-1. As shown in Figure 2(c), decreased HO-1 expression blocked crocin-mediated suppression of LPS-stimulated iNOS expression, whereas transfection with control siRNA had no effect. Moreover, increased HO-1 expression by an inducer of HO-1 (CoPP) suppressed LPS-stimulated iNOS expression (Figure 2(d)). These results suggest that HO-1 induced by crocin suppresses the expression of iNOS.

**Figure 3:**
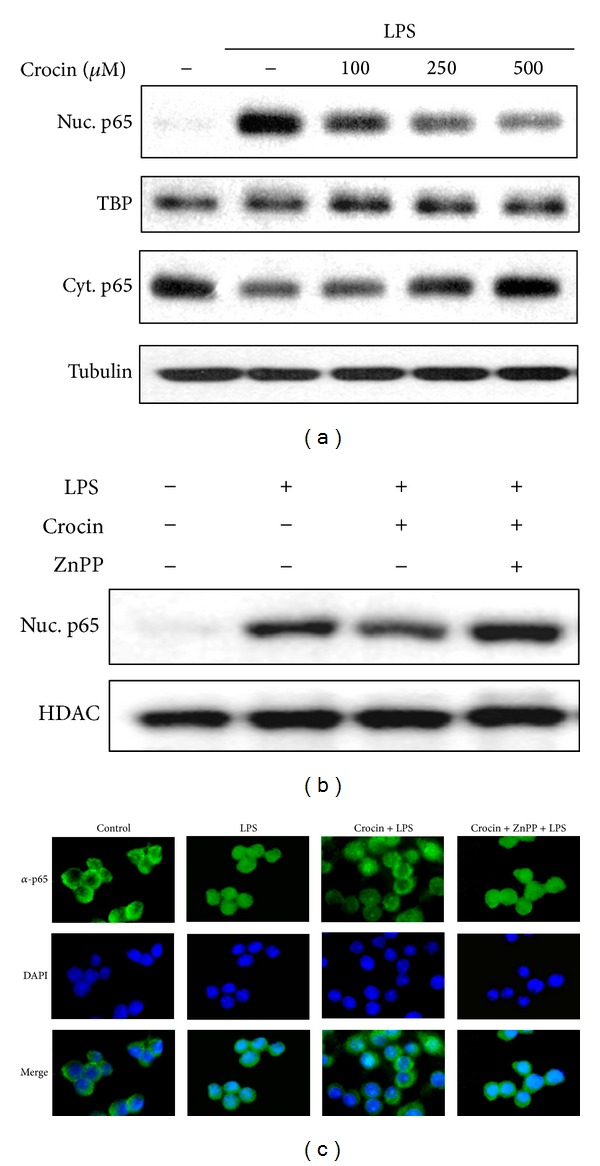
Inhibitory effects of crocin on NF-*κ*B translocation in LPS-stimulated macrophages. (a) Cells were incubated with the indicated concentrations of crocin for 3 h and then stimulated with LPS (0.1 *μ*g/mL) for 30 min, after which nuclear and cytosolic proteins were analyzed by Western blotting. (b) Cells were treated with crocin (500 *μ*M) in the absence or presence of ZnPP (1 *μ*M) for 3 h and then stimulated with LPS (0.1 *μ*g/mL) for 30 min, after which nuclear proteins were analyzed by Western blotting. (c) Cells were incubated with crocin in the absence or presence of ZnPP as described above and then stimulated with LPS (0.1 *μ*g/mL) for 30 min. Fixed cells were subsequently stained with anti-NF-*κ*B, FITC-conjugated anti-rabbit IgG antibody, and DAPI, and images were taken using fluorescence microscope.

Since iNOS promoter which contains two binding sites for transcription factor NF-*κ*B and NF-*κ*B plays a critical role in LPS-induced iNOS expression, we investigated whether crocin suppresses iNOS expression via the regulation of NF-*κ*B activity. Crocin inhibited the translocation of NF-*κ*B p65 from cytosol into nucleus in a dose-dependent manner (Figure 3(a)). Additionally, crocin dose-dependently suppressed LPS-stimulated binding of NF-*κ*B to DNA and I*κ*B-*α* degradation (Supplementary Figures 2(A) and 2(B)). The involvement of HO-1 in NF-*κ*B activation was tested using a specific HO competitive inhibitor, zinc protoporphyrin IX (ZnPP) [25]. Pretreatment with ZnPP attenuated crocin-mediated suppression of NF-*κ*B translocation (Figure 3(b)). NF-*κ*B translocation was confirmed by immunofluorescence microscopy. Crocin attenuated LPS-mediated nuclear translocation of NF-*κ*B p65 in RAW 264.7 cells, while crocin-mediated suppression of NF-*κ*B translocation was obstructed by HO-1 inhibitor ZnPP (Figure 3(c)). These results suggest that crocin inhibits iNOS expression and NF-*κ*B activation through the induction of HO-1 in macrophages.

### 3.3. Nrf2 Is Activated by Crocin in Macrophages

**Figure 4:**
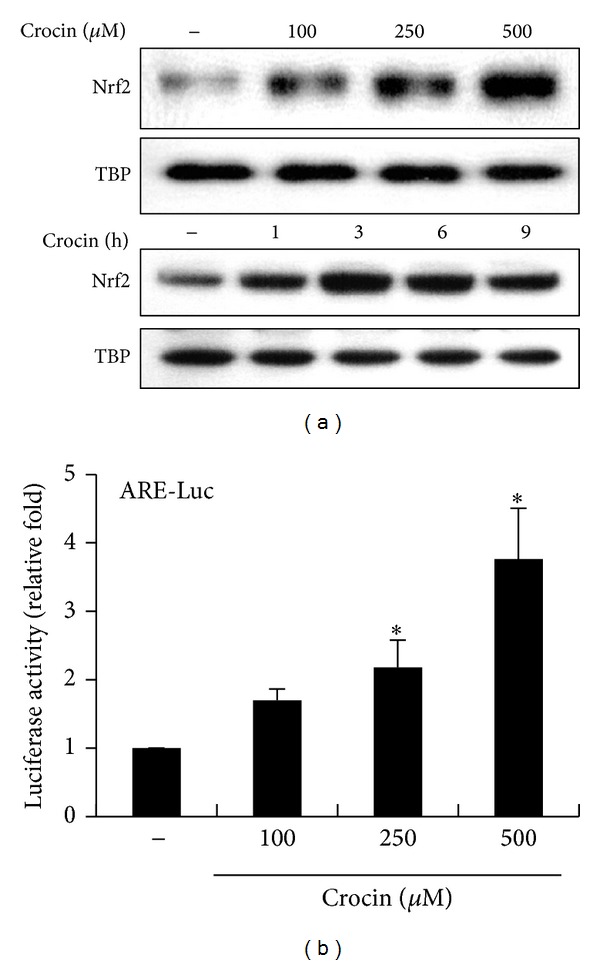
Activation of Nrf2 by crocin. (a) RAW 264.7 cells were incubated with the indicated concentrations of crocin for 3 h (upper panel) or with 500 *μ*M crocin for various periods (lower panel), after which nuclear extracts were harvested and assayed by Western blotting. TATA binding protein (TBP) was used as a loading control. (b) Cells were transfected with ARE-luciferase construct and then treated with the indicated concentration of crocin. Equal amounts of cell extracts were then assayed for dual luciferase activity. *P < 0.05 versus the group treated with PBS (vehicle).

Since the promoter region of the* ho-1* gene contains binding sites for transcription factor Nrf2 and the expression of HO-1 is known to be regulated by Nrf2, we determined whether crocin activates Nrf2 in RAW 264.7 cells. Nrf2 nuclear translocation, which is critical to its transcriptional activity, increased in response to crocin in a dose-dependent manner, reaching its peak at 3 h (Figure 4(a)). Crocin also induced binding of Nrf2 to the antioxidant response element (ARE) sequence (Supplementary Figure 3). To elucidate the effects of crocin on transactivation activity of Nrf2, RAW 264.7 cells were transiently transfected with luciferase reporter genes driven by ARE to which Nrf2 binds. As shown in Figure 4(b), crocin treatment increased ARE promoter activity in a dose-dependent manner. Taken together, these results suggest that crocin activates Nrf2, which then induces HO-1 expression.

### 3.4. Crocin Induces HO-1 Expression via Mobilization of Intracellular Free Ca^2+^

**Figure 5:**
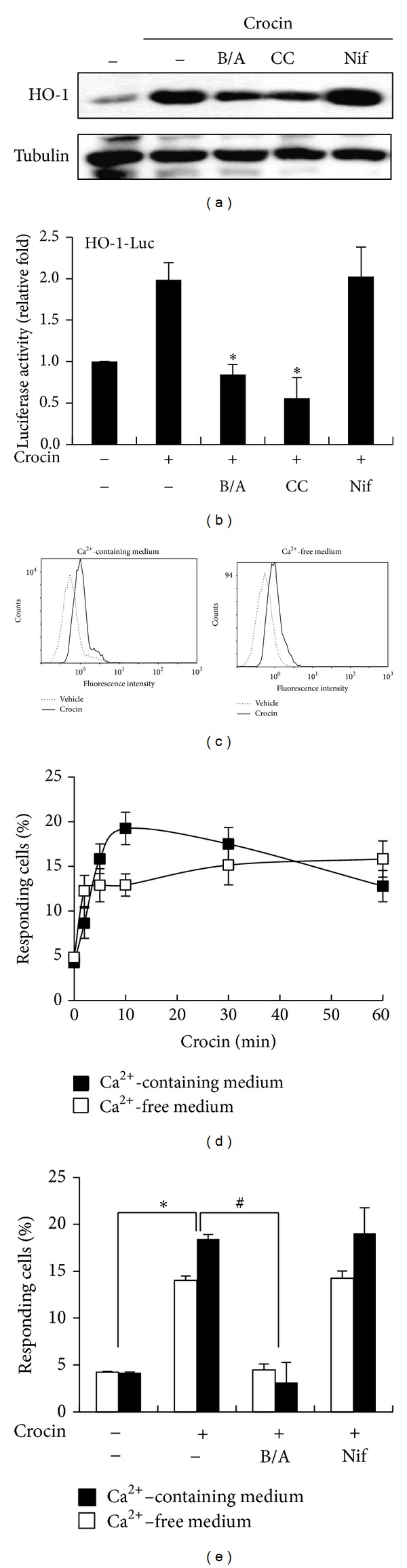
Induction of HO-1 expression via elevation of intracellular Ca^2+^ levels. (a) RAW 264.7 cells were incubated with BAPTA/AM (B/A, 5 *μ*M), calmidazolium chloride (CC, 5 *μ*M), and nifedipine (Nif, 5 *μ*M) for 1 h and then treated with crocin (500 *μ*M) for 6 h. Equal amounts of cytosolic proteins were subsequently analyzed by Western blotting with HO-1 antibody. Tubulin was used as a loading control. (b) Cells were transfected with HO-1 promoter-luciferase construct and then treated as described above, after which equal amounts of cell extracts were assayed for dual luciferase activity. *P < 0.05 versus the group treated with crocin alone. (c) Intracellular free Ca^2+^ levels were assessed by flow cytometry. Cells were treated with vehicle or crocin (500 *μ*M) for 10 min in either Ca^2+^-containing medium (left) or Ca^2+^-free medium (right). (d) The changes of intracellular Ca^2+^ levels with time were represented as percentages of responding cells. (e) Cells were treated with crocin (500 *μ*M) in the absence or presence of BAPTA/AM (B/A, 5 *μ*M), or nifedipine (Nif, 5 *μ*M) for 10 min, after which intracellular free Ca^2+^ levels (represented as percentage of responding cells) were evaluated by flow cytometry. *P < 0.05 versus the group treated with PBS (vehicle); ^#^ *P* < 0.05 versus the group treated with crocin alone in both Ca^2+^-containing and Ca^2+^-free medium.

To investigate the signaling pathways associated with crocin-induced HO-1 expression, we examined the effects of several inhibitors related to Ca^2+^ signaling including BAPTA/AM, the intracellular Ca^2+^ chelator, calmidazolium chloride, the calmodulin antagonist, and nifedipine, the L-type Ca^2+^ channel blocker. HO-1 expression and HO-1 promoter activity were inhibited by BAPTA/AM and calmidazolium chloride but not by nifedipine (Figures 5(a) and 5(b)), suggesting that intracellular Ca^2+^/calmodulin is critical to crocin-mediated HO-1 expression. We next examined whether crocin triggers the release of Ca^2+^ from intracellular pools. To accomplish this, RAW 264.7 cells were loaded with Fluo-3/AM and the level of intracellular free Ca^2+^ was measured by FACS in Ca^2+^-containing or Ca^2+^-free medium. As shown in Figures 5(c) and 5(d), crocin treatment resulted in an increase in [Ca^2+^]_i_ in either Ca^2+^-containing or Ca^2+^-free medium. Moreover, the crocin-mediated increase in [Ca^2+^]_i_ was inhibited by BAPTA/AM but not by nifedipine (Figure 5(e)), indicating that crocin induces Ca^2+^ mobilization from intracellular pools. Taken together, these results suggest that crocin-mediated HO-1 expression is triggered by Ca^2+^ efflux from intracellular stores rather than Ca^2+^ influx from extracellular fluid.

### 3.5. Ca^2+^/Calmodulin-Dependent Kinase 4 (CAMK4) Mediates Crocin-Induced HO-1 Expression

**Figure 6:**
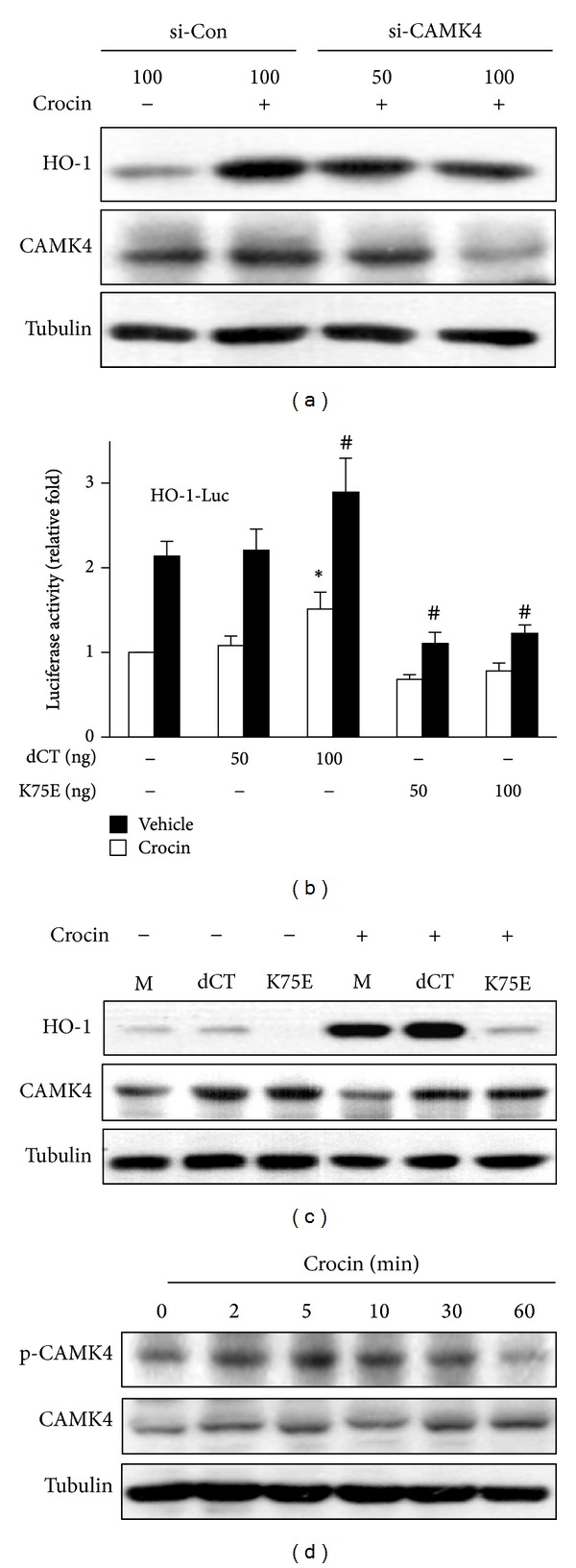
Involvement of CAMK4 in crocin-mediated expression of HO-1. (a) RAW 264.7 cells were transfected with CAMK4 siRNA or control siRNA and then treated with crocin (500 *μ*M) for 6 h. Equal amounts of cytosolic proteins were analyzed by Western blotting. (b) Cells were cotransfected with HO-1 promoter-luciferase construct and pcDNA3.1, CAMK4 constitutive active (dCT), or kinase dead (K75E) construct and then treated with crocin (500 *μ*M). Equal amounts of cell extracts were assayed for dual luciferase activity. *P < 0.05 versus the pcDNA3.1-transfected (vehicle-treated) group; ^#^ *P* < 0.05 versus the pcDNA3.1-transfected (crocin-treated) group. (c) Cells were transfected with pcDNA3.1 (mock, M), dCT or K75E construct and then treated with crocin (500 *μ*M). Equal amounts of cytosolic proteins were analyzed by Western blotting. (d) Cells were treated with crocin (500 *μ*M) for the indicated time and the levels of phosphorylated CAMK4 and total CAMK4 were analyzed by Western blotting. Tubulin was used as a loading control.

Ca^2+^ is an important second messenger and several signaling molecules are regulated by Ca^2+^/calmodulin, among which CAMK is a common target. We investigated whether CAMK4 is associated with crocin-induced HO-1 expression using siRNA because no specific inhibitor of CAMK4 was found. As shown in Figure 6(a), crocin-induced HO-1 expression was suppressed by CAMK4 knockdown. Next, we examined the effects of ectopic expression of CAMK4 mutant on HO-1 expression. Transient transfection of RAW 264.7 cells with catalytically inactive CAMK4 mutant (K75E) inhibited crocin-induced activation of HO-1 promoter and HO-1 expression, while constitutively active CAMK4 mutant (c-terminal autoinhibitory domain-deletion construct, dCT) slightly increased these factors (Figures 6(b) and 6(c)). Moreover, crocin induced rapid phosphorylation of CAMK4, which peaked at 5 min and then declined to basal levels within 60 min (Figure 6(d)). These results suggest that activation of CAMK4 is involved in crocin-mediated HO-1 expression.

### 3.6. CAMK4 Mediates Crocin-Induced Nrf2 Activation

**Figure 7:**
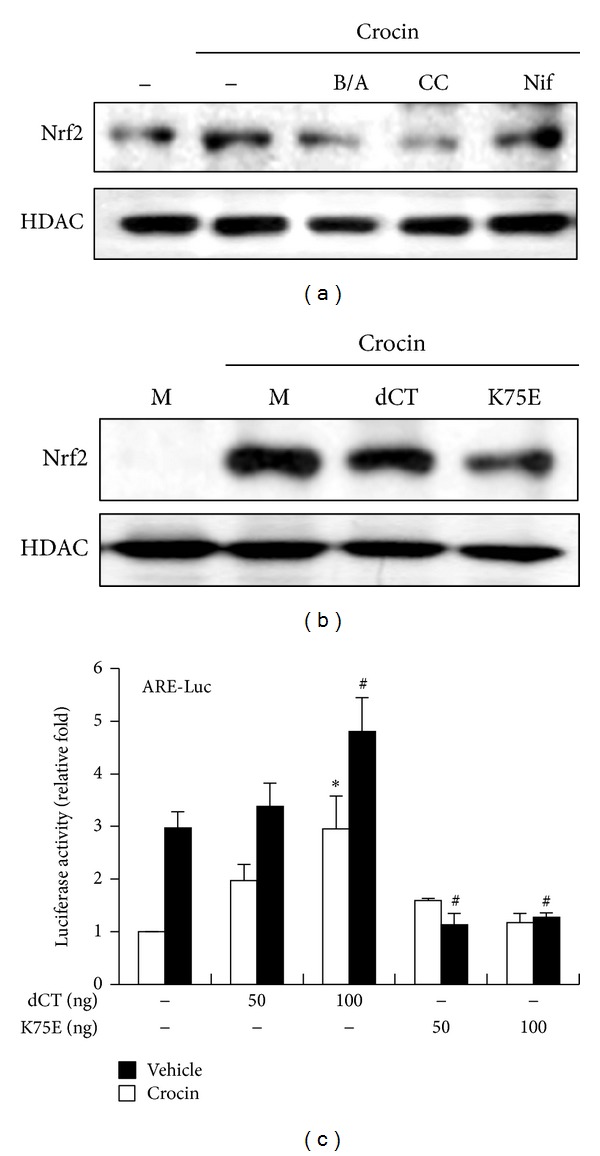
Regulation of Nrf2 activation by CAMK4. (a) RAW 264.7 cells were incubated with BAPTA/AM (B/A, 5 *μ*M), calmidazolium chloride (CC, 5 *μ*M), and nifedipine (Nif, 5 *μ*M) for 1 h and then treated with crocin (500 *μ*M) for 3 h. Equal amounts of nuclear proteins were subsequently analyzed by Western blotting. (b) Cells were transfected with pcDNA3.1 (mock, M), CAMK4 constitutive active (dCT), or kinase dead (K75E) construct and then treated with crocin (500 *μ*M). Equal amounts of nuclear proteins were then analyzed by Western blotting. HDAC was used as a loading control. (c) Cells were cotransfected with ARE-luciferase construct and pcDNA3.1, dCT, or K75E and then treated with crocin (500 *μ*M). Equal amounts of cell extracts were assayed for dual luciferase activity. *P < 0.05 versus the pcDNA3.1-transfected (vehicle-treated) group; ^#^ *P* < 0.05 versus the pcDNA3.1-transfected (crocin-treated) group.

Since expression of HO-1 is regulated by Nrf2 and crocin induced nuclear translocation of Nrf2 and ARE promoter activity, we examined whether Ca^2+^ signaling involved crocin-mediated Nrf2 activation using several inhibitors related to Ca^2+^ signaling. As shown in Figure 7(a), Nrf2 nuclear translocation was inhibited by BAPTA/AM and calmidazolium chloride but not by nifedipine. These findings are consistent with those of HO-1 and indicate that crocin-mediated Nrf2 nuclear translocation is triggered by Ca^2+^ efflux from intracellular stores. Next, to investigate whether CAMK4 mediates crocin-induced Nrf2 activation, the effects of ectopic expression of CAMK4 mutants were examined. Transient transfection of RAW 264.7 cells with catalytically inactive CAMK4 mutant (K75E) inhibited crocin-induced Nrf2 nuclear translocation (Figure 7(b)). Moreover, K75E mutant inhibited crocin-mediated ARE promoter activity, while constitutively active CAMK4 mutant (dCT) slightly increased ARE promoter activity (Figure 7(c)). These results suggest that CAMK4 mediates crocin-induced Nrf2 activation which contributes to the upregulation of HO-1 by crocin.

### 3.7. Akt Acts Downstream of CAMK4 during Crocin-Induced HO-1 Expression

**Figure 8:**
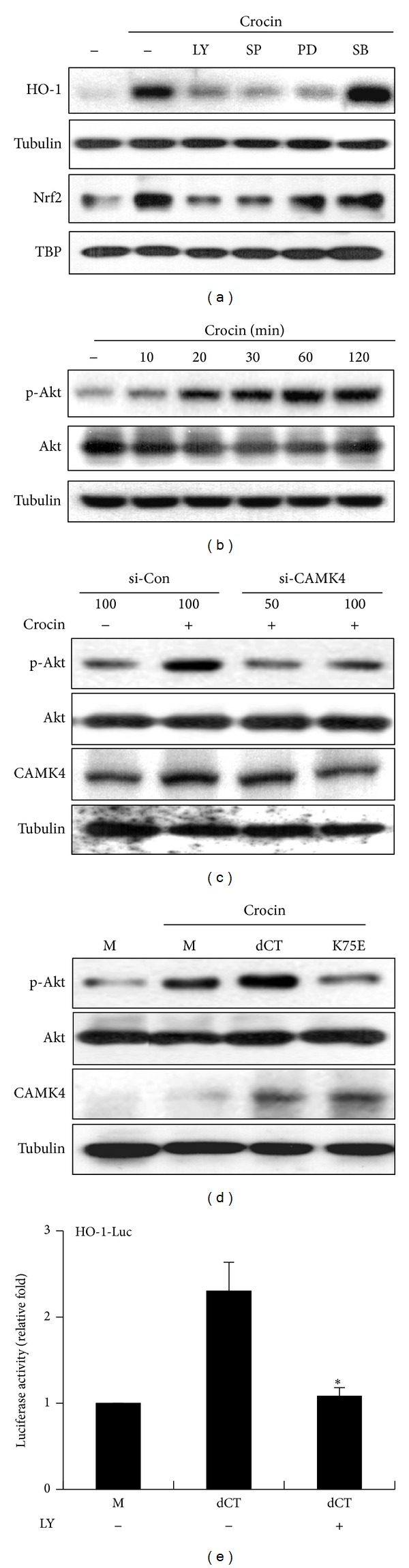
Akt acts as a downstream regulator of CAMK4 in crocin-mediated HO-1 expression. (a) RAW 264.7 cells were incubated with LY294002 (20 *μ*M), SP600125 (20 *μ*M), PD98059 (20 *μ*M), and SB203580 (20 *μ*M) for 1 h and then treated with crocin (500 *μ*M) for 6 h. Equal amounts of cytosolic and nuclear extract were subsequently analyzed by Western blotting with HO-1 and Nrf2 antibody, respectively. Tubulin and TBP were used as loading controls. (b) Cells were incubated with crocin (500 *μ*M) for the indicated times. (c) Cells were transfected with CAMK4 siRNA or control siRNA and then treated with crocin (500 *μ*M) for 1 h. (d) Cells were transfected with pcDNA3.1 (mock, M), CAMK4 constitutive active (dCT), or kinase dead (K75E) construct and then treated with crocin (500 *μ*M) for 1 h, after which equal amounts of cytosolic extract were analyzed by Western blotting. (e) Cells were cotransfected with HO-1-luciferase construct and pcDNA3.1, or dCT and then treated with LY294002 (20 *μ*M). Equal amounts of cell extracts were then assayed for dual luciferase activity. *P < 0.05 versus the dCT-transfected (no LY294002) group.

Since PI3K/Akt and three MAPKs (ERK1/2, JNK, and p38 MAPK) have been reported to be involved in HO-1 expression in response to diverse stimuli [1], we investigated whether CAMK4 is associated with these signaling pathways in crocin-induced HO-1 expression. To accomplish this, we examined which signaling pathway regulates crocin-mediated expression of HO-1 using the pharmaceutical protein kinase inhibitors, LY294002 (PI3K inhibitor), SP600125 (JNK inhibitor), PD98059 (ERK1/2 inhibitor), and SB203580 (p38 MAPK inhibitor). As shown in Figure 8(a), crocin-induced HO-1 expression and Nrf2 nuclear translocation were significantly inhibited by LY294002, SP600125, and PD98059 but not by SB203580. Moreover, crocin increased the phosphorylation of Akt (Figure 8(b)), ERK, and JNK, although ERK and JNK were slightly phosphorylated by crocin (Supplementary Figure 4(A)). These results indicate that PI3K/Akt, ERK, and JNK signalings occur upstream of crocin-mediated HO-1 expression. Next, to determine the relationship of CAMK4 and these kinases during crocin-induced HO-1 expression, the effects of CAMK4 inhibition on the activities of these kinases were examined. Western blot analysis showed that crocin-induced Akt activation was suppressed by CAMK4 knockdown (Figure 8(c)) and catalytically inactive CAMK4 mutant (Figure 8(d)), but ERK and JNK activation were not influenced (Supplementary Figures 4(B) and 4(C)). Moreover, increased HO-1 promoter activity by constitutively active CAMK4 mutant was significantly reduced following treatment with LY294002 (Figure 8(e)). These results suggest that CAMK4 acts as an upstream regulator of PI3K/Akt in crocin-mediated HO-1 expression.

### 3.8. CAMK4 Is Required for Crocin-Mediated Inhibition of iNOS Expression

**Figure 9:**
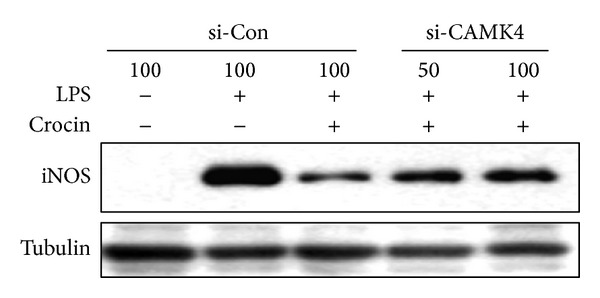
CAMK4 is necessary for crocin-mediated inhibition of iNOS expression in LPS-stimulated macrophages. RAW 264.7 cells were transfected with CAMK4 siRNA or control siRNA and then treated with crocin (500 *μ*M). After 3 h, cells were incubated with LPS (0.1 *μ*g/mL) for 24 h. Equal amounts of cytosolic extract were analyzed by Western blotting. Tubulin was used as a loading control.

To determine whether CAMK4 contributes to the inhibitory effects of crocin on iNOS expression, cells were treated with CAMK4 siRNA and iNOS expression was examined. As shown in Figure 9, CAMK4 knockdown rescued crocin-mediated inhibition of iNOS expression in LPS-stimulated macrophages. These results suggest that CAMK4 plays a pivotal role in crocin-mediated suppression of iNOS expression in LPS-stimulated macrophages.

## 4. Discussion

HO-1 is believed to exhibit anti-inflammatory activities by inhibiting production of proinflammatory mediators in a variety of cells [26–28]. However, the precise mechanism by which HO-1 exerts its anti-inflammatory effects has yet to be fully elucidated. The results of the present study demonstrate that crocin inhibited LPS-stimulated iNOS expression by inducing HO-1 in macrophages providing the first evidence of the pivotal roles of CAMK4 in crocin-induced expression of HO-1.

We found that crocin markedly induced HO-1 expression at the transcriptional level in the murine macrophage cell line, RAW 264.7, as well as in mouse peritoneal macrophages. Because HO-1 is known to be induced by chemical-mediated oxidative stress, we examined whether crocin increased HO-1 expression by inducing cell damage. A cell viability assay (MTT assay) revealed that crocin does not affect cell viability, suggesting that HO-1 is specifically induced by crocin, not by cell damage. Next, we examined whether HO-1 expression is correlated with the inhibition of LPS-induced iNOS expression. Crocin significantly inhibited iNOS expression and NO production in LPS-stimulated macrophages, while the knockdown of HO-1 expression by siRNA markedly reversed the inhibitory effects of crocin on iNOS expression in LPS-induced macrophages. These data indicate that crocin inhibits iNOS expression via the modulation of HO-1 expression and are consistent with other reports which demonstrated the induction of HO-1 expression plays a significant role in mediating the anti-inflammatory effects of various natural compounds in LPS-stimulated macrophages. Although the contribution of HO-1 products (i.e., CO, biliverdin, and iron) was not examined in this study, several studies point to HO-1-derived CO and biliverdin as a potential metabolite to combat inflammation [29–32]. In particular, recent reports demonstrated that biliverdin administration protects against endotoxin-induced acute lung injury in rats [32], and CO and biliverdin ameliorate murine collagen induced arthritis [29]. Taken together, these results suggest that crocin could be useful as a therapeutic agent for many kinds of inflammatory diseases.

Since dysregulation of NF-*κ*B function is associated with inflammation, the development of drugs that control NF-*κ*B is one of promising strategies for treatment of various inflammatory diseases [33–35]. The results of the present study showed that crocin significantly inhibited the translocation of NF-*κ*B p65 subunit into the nucleus and its binding activity to *κ*B-binding motifs on DNA (Supplementary Figure 2). Crocin also suppressed the degradation of I*κ*B-*α* in LPS-stimulated macrophages (Supplementary Figure 2). Additionally, a specific HO competitive inhibitor, ZnPP, blocked crocin-mediated suppression of LPS-induced NF-*κ*B translocation. These results indicate that NF-*κ*B is inhibited by crocin-induced HO-1, which consequently attenuates the expression of iNOS. Because NF-*κ*B regulates many genes involved in inflammation, crocin might inhibit the expression of inflammatory mediators such as TNF-*α*, IL-6, and IL-1*β*, although the effects of crocin on cytokine production have to be confirmed in macrophages.

Despite its role as a potent anti-inflammatory mediator, the intracellular signaling pathway leading to the induction of HO-1 expression is not well understood. Our study showed that crocin mobilized Ca^2+^ from intracellular stores and that crocin-induced expression of HO-1 was dependent on intracellular Ca^2+^/calmodulin. This role of Ca^2+^ signaling in regulation of HO-1 expression has also been observed in several types of cells treated with other compounds such as tertiary-butylhydroquinone, nicotine, and curcumin, suggesting that Ca^2+^-linked signaling could be a common regulatory mechanism of HO-1 expression [36–39]. One of the common targets of Ca^2+^-mediated signaling is CAMK. Kim et al. [36] reported that bisdemethoxycurcumin, an analog of curcumin, mediates induction of HO-1 expression via a Ca^2+^/CAMK2/ERK cascade. However, in the present study, crocin-induced HO-1 expression was not suppressed by CAMK2 inhibitors such as KN62 and KN93 (Supplementary Figure 5). Instead, it was markedly inhibited by CAMK4 siRNA or kinase-dead mutant. Although KN62 and KN93 are known to inhibit CAMK4 activity as well as CAMK2, HO-1 expression was not inhibited by these inhibitors. This might have been due to differences in the sensitivity of pharmacological inhibitors to each CAMK or the unknown side effects of these inhibitors. Because we did not examine the effects of CAMK2 siRNA or mutant, we cannot exclude the possibility that CAMK2 is involved in crocin-induced HO-1 expression. However, the results derived from more specific inhibition of CAMK4 with siRNA and mutant suggest that the Ca^2+^/calmodulin-CAMK4-linked pathway regulates crocin-mediated expression of HO-1.

Nrf2, a bZIP transcription factor, is an essential ARE-binding factor involved in expression of the* ho-1* gene. In this study, Nrf2 activation by crocin was dependent on Ca^2+^/calmodulin-CAMK4. Previous studies demonstrated that PI3K and MAPKs such as ERK, JNK, and p38 participate in the induction of Nrf2/ARE-mediated HO-1 expression in response to various stimuli [40, 41]. However, these signaling pathways depend on the type of cells and stimuli in terms of their contribution to HO-1 expression. Crocin-mediated Nrf2 activation and HO-1 expression were regulated by PI3k/Akt, ERK, and JNK. Moreover, use of siRNA or kinase-dead mutant for CAMK4 showed that CAMK4 acts upstream of Akt and that activation of ERK and JNK is independent of CAMK4. These data demonstrate that, although necessary for the optimal expression of HO-1 by crocin, CAMK4-Akt and MAPKs (ERK and JNK) are activated independently.

In summary, the results of this study demonstrate that crocin induces HO-1 expression though a rapid elevation of [Ca^2+^]_i_ and subsequent activation of CAMK4, Akt, and Nrf2 in macrophages. Furthermore, the results presented herein also demonstrate that crocin-mediated HO-1 inhibits LPS-induced iNOS expression and that knockdown of CAMK4 blocks this suppressive effect on iNOS expression. These findings suggest that crocin exhibits anti-inflammatory effects by upregulation of HO-1 expression via CAMK4-PI3K/Akt-Nrf2 signaling in LPS-stimulated macrophages. Our findings could help elucidate the molecular mechanism of anti-inflammatory action of crocin and suggest that crocin may have therapeutic potential for treatment of inflammatory diseases.

## Supplementary Material

Supplementary Fig. 1: Effects of crocin on HO-1 induction, NO production and iNOS expression in murine peritoneal macrophages.
Supplementary Fig. 2: Inhibitory effects of crocin on DNA binding activity of NF-*κ*B and I*κ*B-*α* degradation in LPS-stimulated macrophages.
Supplementary Fig. 3: Effects of crocin on DNA binding activity of Nrf2.
Supplementary Fig. 4: Effects of crocin and CAMK4 on activity of ERK and JNK.
Supplementary Fig. 5: Effects of CAMK inhibitors on crocin-mediated HO-1 expression.
