# Supplementary material for: Crocin Suppresses LPS-Stimulated Expression of Inducible Nitric Oxide Synthase by Upregulation of Heme Oxygenase-1 via Calcium/Calmodulin-Dependent Protein Kinase 4
Source: Mediators Inflamm. 2014 Apr 15;2014:728709. doi: 10.1155/2014/728709 (PMC4009253; doi:10.1155/2014/728709)

Supplementary Figure legends

Supplementary Fig. 1. Effects of crocin on HO-1 induction, NO production and iNOS expression in murine peritoneal macrophages. TG-elicited mouse peritoneal macrophages were incubated with the indicated concentrations of crocin for 6 h (A) or 500 μM crocin for various periods (B). HO-1 protein levels were analyzed by Western blotting. (C) Cells were incubated with the indicated concentrations of crocin for 3 h and then stimulated with LPS (0.1 μg/ml) for 24 h. The supernatant was subsequently harvested and the amount of nitrite released from cells was measured by the Griess method (A). * $p < 0.05$ vs. the group treated with LPS alone (no crocin). (D) Equal amounts of cytosolic extract in remaining cells were analyzed by Western blotting.

Supplementary Fig. 2. Inhibitory effects of crocin on DNA binding activity of NF-κB and IκB-α degradation in LPS-stimulated macrophages. (A) RAW 264.7 cells were incubated with the indicated concentrations of crocin for 3 h and then stimulated with LPS (0.1 μg/ml) for 30 min, after which nuclear proteins were analyzed by electron mobility shift assay (EMSA) with NF-κB-specific probe. ns, non-specific band; fp, free probe. (B) Cells were treated with the indicated concentrations of crocin for 3 h and then stimulated with LPS (0.1 μg/ml) for 15 min. Equal amounts of cytosolic proteins were analyzed by Western blotting.

Supplementary Fig. 3. Effects of crocin on DNA binding activity of Nrf2. RAW 264.7 cells were incubated with the indicated concentrations of crocin for 3 h, and nuclear proteins were then extracted and analyzed by EMSA with Nrf2-specific probe. ns, non-specific band; fp, free probe.

Supplementary Fig. 4. Effects of crocin and CAMK4 on activity of ERK and JNK. (A) RAW 264.7 cells were incubated with crocin (500 μM) for the indicated times. (B) Cells were transfected with CAMK4 siRNA or control siRNA and then treated with crocin (500 μM) for 1 h. (C) Cells were transfected with pcDNA3.1 (mock, M), CAMK4 constitutive active (dCT) or kinase dead (K75E) construct and then treated with crocin (500 μM) for 1 h. Phosphorylation of ERK and JNK was analyzed by Western blotting.

Supplementary Fig. 5. Effects of CAMK inhibitors on crocin-mediated HO-1 expression. RAW 264.7 cells were incubated with KN62, KN93 or KN92 (10 or 20 μM, respectively) for 1 h and then treated with crocin (500 μM) for 6 h. Equal amounts of cytosolic proteins were then analyzed by Western blotting. The intensity of each band relative to tubulin (fold of control) is indicated in parentheses.

Kim *et al*., Supplementary Fig. 1.

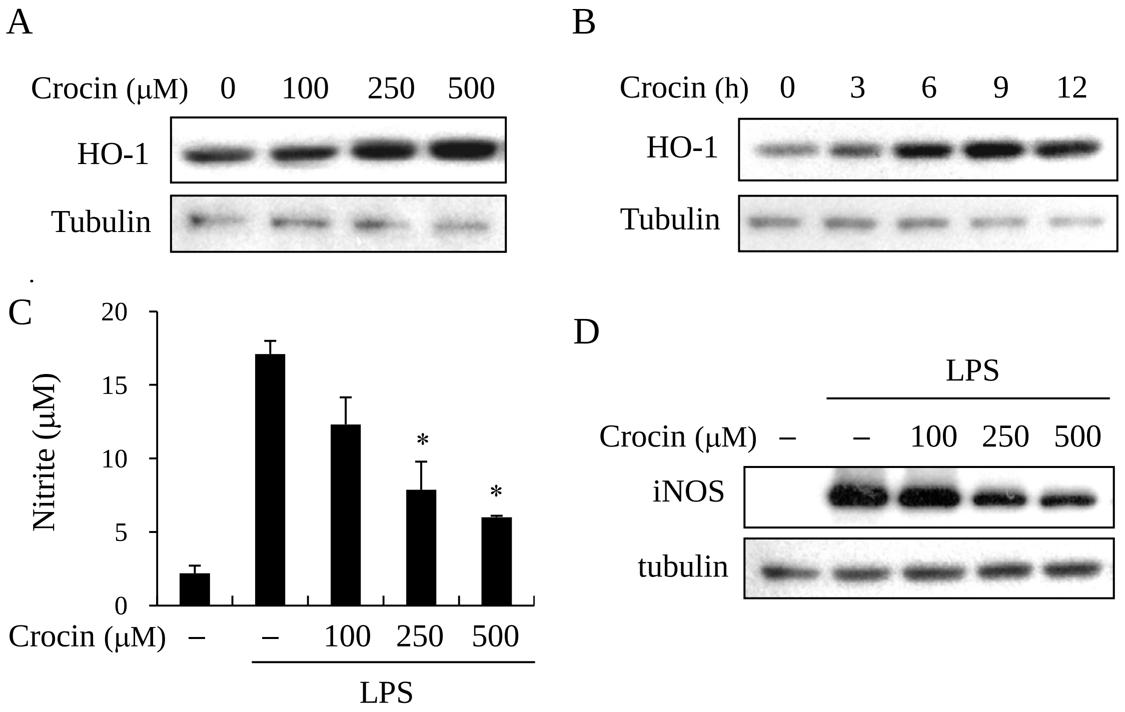

Kim *et al.*, Supplementary Fig. 2

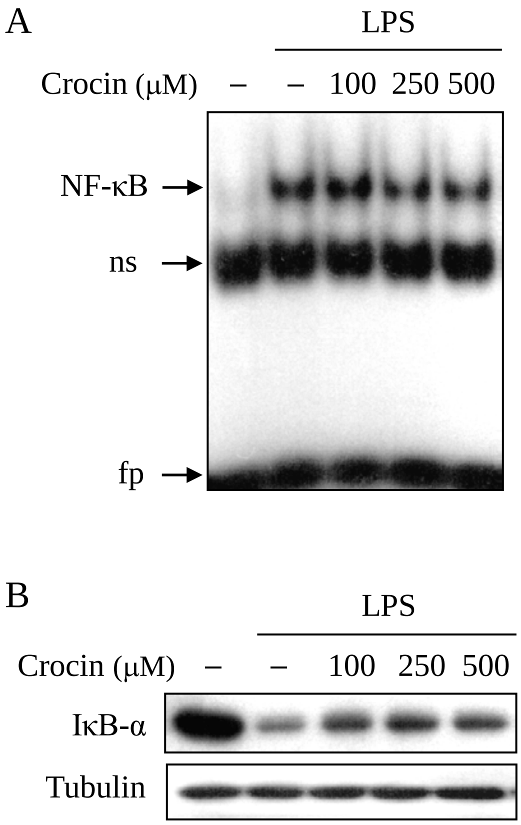

Kim *et al*., Supplementary Fig. 3.

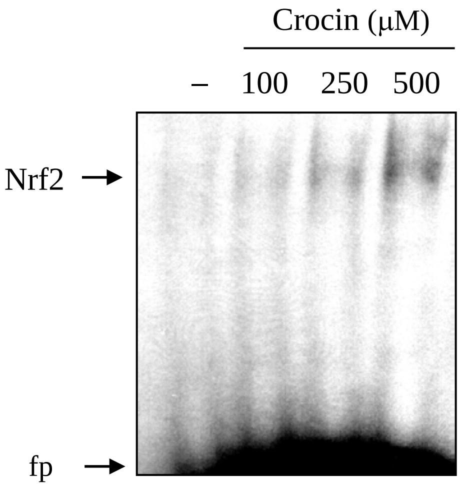

Kim *et al*., Supplementary Fig. 4.

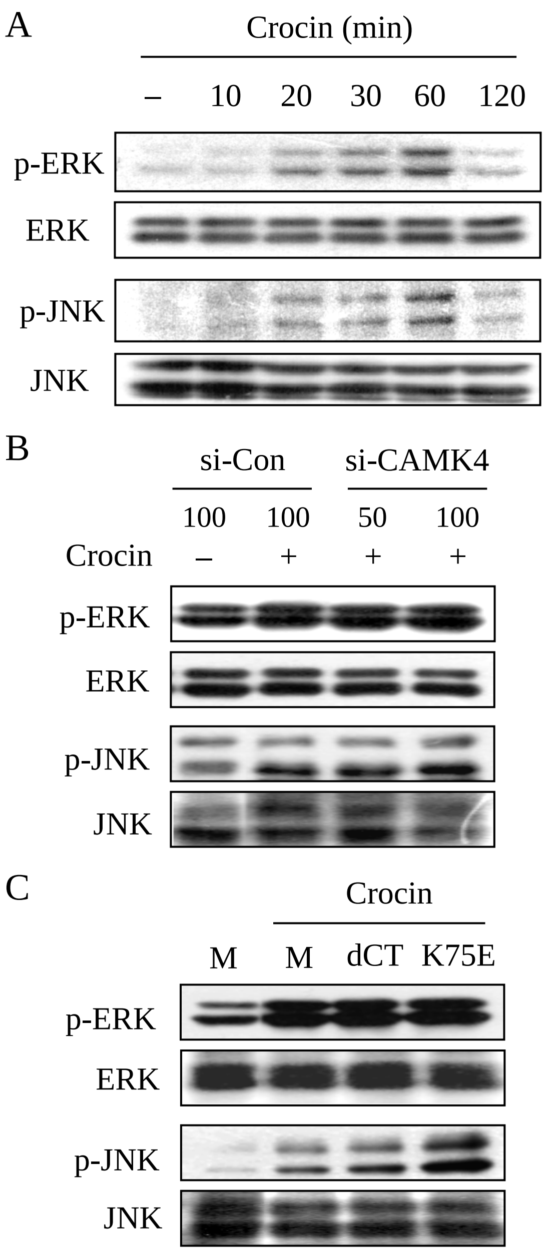

Kim *et al*., Supplementary Fig. 5.

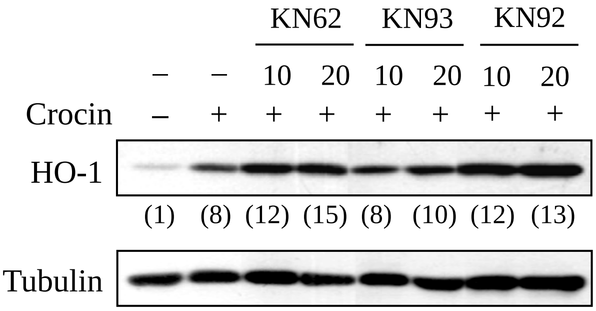

Supplement: Supplementary file 1 — Supplementary Fig. 1: Effects of crocin on HO-1 induction, NO production and iNOS expression in murine peritoneal macrophages. Supplementary Fig. 2: Inhibitory effects of crocin on DNA binding activity of NF-κB and IκB-α degradation in LPS-stimulated macrophages. Supplementary Fig. 3: Effects of crocin on DNA binding activity of Nrf2. Supplementary Fig. 4: Effects of crocin and CAMK4 on activity of ERK and JNK. Supplementary Fig. 5: Effects of CAMK inhibitors on crocin-mediated HO-1 expression. [file 728709.f1.pdf]
